# OSWG Recommended Approaches to the Nonclinical Pharmacokinetic (ADME) Characterization of Therapeutic Oligonucleotides

**DOI:** 10.1089/nat.2023.0011

**Published:** 2023-10-03

**Authors:** Cindy L. Berman, Madeleine Antonsson, Sandor Batkai, Sieto Bosgra, Girish R. Chopda, Wouter Driessen, Jeffrey Foy, Chopie Hassan, Xiao Shelley Hu, Hyun Gyung Jang, Meena, Mark Sanseverino, Thomas Thum, Yanfeng Wang, Martin Wild, Jing-Tao Wu

**Affiliations:** ^1^ Berman Consulting, Wayland, Massachusetts, USA.; ^2^ Early CVRM, Biopharmaceuticals R&D, AstraZeneca, Gothenburg, Sweden.; ^3^ Cardior Pharmaceuticals GmbH, Hannover, Germany.; ^4^ Independent Consultant, Amsterdam, The Netherlands.; ^5^ Dicerna Pharmaceuticals, Inc., a Novo Nordisk Company, Lexington, Massachusetts, USA.; ^6^ Anjarium Biosciences AG, Zurich, Switzerland.; ^7^ PepGen, Inc., Cambridge, Massachusetts, USA.; ^8^ ProQR Therapeutics NV, Leiden, The Netherlands.; ^9^ Wave Life Sciences, Cambridge, Massachusetts, USA.; ^10^ Stoke Therapeutics, Bedford, Massachusetts, USA.; ^11^ Agilent Therapeutics, Boulder, Colorado, USA.; ^12^ Institute of Molecular and Translational Therapeutic Strategies (IMTTS), Hannover, Germany.; ^13^ Fraunhofer Institute of Toxicology and Experimental Medicine, Hannover, Germany.; ^14^ Formerly of Ionis Pharmaceuticals, Carlsbad, California, USA.; ^15^ Early Oncology, Oncology R&D, AstraZeneca, Cambridge, United Kingdom.; ^16^ Alnylam Pharmaceuticals, Cambridge, Massachusetts, USA.

**Keywords:** ADME, ASO, siRNA, OSWG, oligonucleotides, pharmacokinetics

## Abstract

This white paper summarizes the recommendations of the absorption, distribution, metabolism, and excretion (ADME) Subcommittee of the Oligonucleotide Safety Working Group for the characterization of absorption, distribution, metabolism, and excretion of oligonucleotide (ON) therapeutics in nonclinical studies. In general, the recommended approach is similar to that for small molecule drugs. However, some differences in timing and/or scope may be warranted due to the greater consistency of results across ON classes as compared with the diversity among small molecule classes. For some types of studies, a platform-based approach may be appropriate; once sufficient data are available for the platform, presentation of these data should be sufficient to support development of additional ONs of the same platform. These recommendations can serve as a starting point for nonclinical study design and foundation for discussions with regulatory agencies.

## Introduction

This document presents the Subcommittee's recommendations for strategies to assess the nonclinical pharmacokinetics (PK), including absorption, distribution, metabolism, and excretion (ADME) properties of oligonucleotides (ONs). The scope encompasses all ONs, including both single-stranded and double-stranded ONs, regardless of pharmacological mechanism of action, structure, or chemical modifications. In general, the strategies are similar for all types of ONs; however, the recommendations are modified for particular types of ONs (eg, conjugated, formulated) as warranted, and messenger RNA (mRNA) ONs are also addressed. For the most part, the document is based on systemic routes of administration, as this is the case for most ONs in development, but mention is made of any special considerations for those ONs administered locally. This document is not intended as a review of published or unpublished PK/ADME data for ONs; much of this has been covered elsewhere [1]. In addition, technical recommendations regarding bioanalytical methods as well as clinical aspects of PK/ADME assessments were considered out of scope.

Furthermore, the objective was to provide science-based recommendations and raise “points to consider” regarding study designs, timing, and use of the data generated. These recommendations are the consensus opinion of the Subcommittee and do not necessarily reflect the current expectations of regulatory authorities.

## Regulatory Guidance Regarding PK/ADME Testing

In contrast to safety testing, regulatory guidances regarding nonclinical PK/ADME studies of pharmaceuticals are quite limited. A general outline of the timing of nonclinical safety studies relative to clinical development is provided in International Conference on Harmonization (ICH) M3(R2) guidance [2]. ICH S3A guidance [3] provides general information on expectations regarding toxicokinetics (TK); on the other hand, ICH S3B (Pharmacokinetics: Guidance for repeated dose tissues distribution studies) [4] is rarely consulted by either Sponsors or regulatory authorities. Recommendations on metabolite safety testing is provided in the US Food and Drug Administration (FDA) metabolite in safety testing (also known as MIST) guidance [5]. Information on expectations regarding PK drug–drug interaction (DDI) testing is presented in recent guidances by the European Medicines Agency (EMA) [6] and FDA [7]; a new draft FDA guidance [8] provides considerations specific to ONs.

In addition, recommendations relevant to the development of formulated ONs can be found in the FDA guidance on liposome drug products [9]; ICH S9 [10] also contains some helpful information regarding conjugated and liposomal products that is probably more broadly applicable than to just anticancer pharmaceuticals. Although valuable, these guidance documents do not cover all aspects of PK/ADME and do not provide as much detail as included in those regarding safety testing. Therefore, Sponsors have minimal direction, particularly for relatively new drug classes such as therapeutic ONs. Furthermore, these guidance documents are not applicable to mRNA therapeutics, which, from a regulatory perspective, are regarded as advanced therapy medicinal products (ATMP); the guidances on gene therapy products [11,12] are applicable in Europe and the United States, respectively. For these reasons, the Subcommittee considered and has made recommendations of appropriate strategies for each aspect of PK/ADME testing.

The Subcommittee based its recommendations not only on the information in these guidances, but also on regulatory authority feedback regarding nonclinical ADME for ON programs in development, and nonclinical data required to support clinical development. Although historical experience, such as expectations for small molecule drugs and biologics, and experience with approved ONs, was also considered, the objective was to propose “best practices” for the future based on the unique chemical characteristics of ONs and the current database on their ADME profiles.

## General Considerations for Nonclinical Study Design

In general, the PK/ADME study design considerations for ONs are similar to those for other classes of drugs. The approaches used to select dose levels and the route of administration (ROA) for nonclinical studies of ONs should be no different from those used for small molecule drugs administered by the same ROA. The appropriate group size for PK/ADME studies is study specific. Specific commentary about group sizes is included in some of the following subsections, when warranted.

With regard to definition of the test article, the Subcommittee recommends that the drug product or comparable formulation be tested. That is, for greatest clinical relevance, conjugated or formulated ONs should be administered as such, unless otherwise specified for certain platform-based approaches. However, early in development, “unformulated” ONs can be administered in simple solutions until the drug product is defined. Sponsors should have a clear understanding of the impurity profile of the drug substance to differentiate between shortmers existing in the drug versus metabolites formed *in vivo*.

**FIG. 1.**
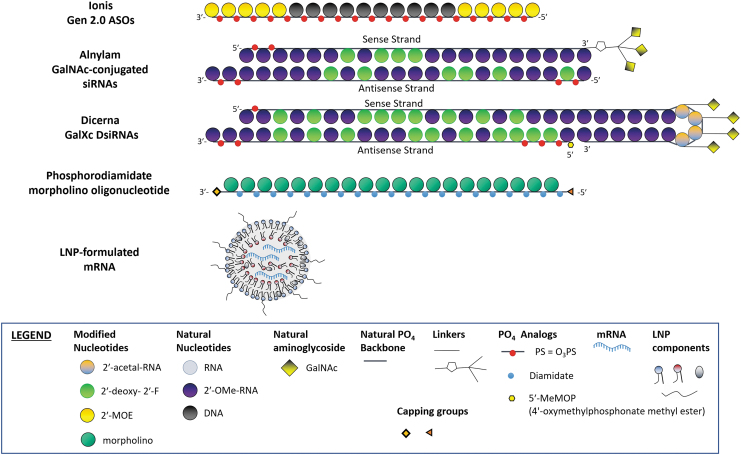
Common oligonucleotide platforms.

When using a platform-based approach, the platform should be defined clearly with regard to the combination of structure, length, backbone chemistry, chemical modifications, conjugate, and/or delivery formulation. The chemical composition of the FDA-approved ONs has been described by Roberts *et al.* [13]. In addition, ONs within a platform should have the same pattern of substitutions and chemistry and similar physiochemical properties and ADME profiles. Thus, the definition of each platform needs to be clear, narrow, and supported by data. Examples (Fig. 1) of platforms include:

- Ionis' generation 2.0/2.5 antisense ONs: short single-stranded ONs, generally 16–20 nucleotides in length with phosphorothioate (PS) linkages, with either (1) 2′-O-(2-methoxyethyl) (2′-MOE) for generation 2.0 [or 2′-O, 4′-C-[(S)-ethylidene] (constrained ethyl, or 2′-cEt) modifications for generation 2.5] at both ends, flanking a central DNA region (gap) that supports the RNase H mechanism in degradation of the target RNA, or (2) full 2′-MOE modifications of the molecule to modulate target RNA splicing. An N-acetylgalactosamine (GalNAc) moiety may be attached to either the 5′- or 3′-end of the antisense oligonucleotides (ASOs), to improve delivery to hepatocytes. Other companies are developing chimeric ASOs flanked with conformationally constrained (ie, locked) nucleic acids (LNAs) [14,15].
- Alnylam's GalNAc-conjugated small interfering RNAs (siRNAs) with enhanced stabilization chemistry (ESC): siRNAs that are fully modified with various chemical modifications, such as 2′-F, 2′-O-methyl, and PS linkages, with optimized placement to maximize metabolic stability while maintaining high intrinsic potency and GalNAc moieties attached to 3′-end of the sense strand.
- Dicerna's GalXC™ DsiRNAs: siRNAs that are fully chemically modified with 2′-F, 2′-O-methyl, and PS linkages, and have a common tetraloop on the sense strand where the GalNAc moieties are attached.
- Phosphorodiamidate morpholino oligonucleotides (PMOs) are single-stranded DNA analogs with a backbone of morpholine rings with phosphorodiamidate linkages. PMOs are charge neutral.
- Lipid nanoparticle (LNP)-formulated mRNA platform: various mRNA molecules consisting of native nucleotides and formulated in the same LNP, which comprise novel and non-novel components in well-defined ratios and particle size. For each LNP platform, the specific components and their ratios must remain constant.

The Subcommittee does not recommend any specific bioanalytical methods to support ADME characterization. A Sponsor should have sound scientific rationale for the methods developed, for instance, to measure parent molecule with and/or without conjugated ligand, and with and/or without potential metabolites. For duplex siRNAs, analysis of the antisense strand is considered representative of the sense strand because, if not annealed, both strands are labile.

Inclusion of species-specific surrogates in certain toxicity studies may be necessary to evaluate effects related to pharmacologic activity in at least one species. However, PK/ADME characterization of an animal-active surrogate is not a suitable substitute for assessment of the clinical candidate. Confirmation of exposure to the surrogate can often be achieved through demonstration of pharmacodynamic (PD) activity rather than PK/TK assessments.

In selecting the species for ON PK/ADME studies, the Subcommittee recommends that the following points be taken into consideration.

(1) The species used for other aspects of the nonclinical development program. Use of the same species for pharmacology and toxicity evaluations provides continuity and allows the possibility of a more integrated, mechanistic understanding of PK/toxicity/PD relationships.
(2) The similarity in target distribution between the species and humans. Ideally, the species selected should mimic the target distribution in humans, if known, to provide the most clinically relevant results. However, this information is often not available, especially at an early stage in development. If targeting moieties are employed, an assessment of the ability of the ON to target the relevant animal tissue should be provided to support the relevance of the species.
(3) The use of alternative nonrodent species for screening. In line with the desire to reduce reliance on monkeys [16,17], Sponsors should consider the use of alternative nonrodent species (dogs and/or minipigs) for early PK screening studies in which PD activity is not a requirement. However, after lead selection, it may be desirable to switch to a pharmacologically responsive species for continuity with the toxicity program.

Metabolism is only a minor consideration because it is generally consistent across species. For most ON modalities, monkeys are selected as the nonrodent species in part because their PK is regarded as predictive of humans; rodents are less predictive due to their more rapid clearance. Regardless of the species chosen, the rationale for the selection, as well as the limitations, should be included in the regulatory submission.

## Absorption

The absorption and PK of a new molecular entity (NME) are normally studied in stand-alone animal pharmacology and/or toxicity studies to establish dose–exposure relationship at both pharmacologically relevant and toxic dose levels to support first-in-human (FIH) studies. Such support includes projection of human pharmacologically active and/or efficacious dose, starting dose, and washout period in Phase 1 studies. In many aspects, the objectives, design principles, and data analyses for ONs do not differ from conventional practice for small molecules and biologics, although special considerations for ONs are emphasized here whenever applicable. Given their unique physicochemical properties, such as size, charge, and hydrophilicity, ON drugs are administered either systemically using parenteral routes, such as intravenous (IV) or subcutaneous (SC) injections, or locally, such as intrathecal (IT) or intracerebroventricular (ICV) injection, inhalation, or intravitreal (IVT) injection. The intended route of clinical administration should be used in animal studies to ensure comparable distribution between animals and humans.

### Single-dose PK study

A single-dose PK study is often conducted, although not required, but the data will add value in understanding the ADME profile of the NME. For ON molecules with new chemistry, when the understanding of PK properties is inadequate, a stand-alone single-dose PK study is recommended. Such a study helps to characterize the PK terminal phase, establishes a correlation between plasma and/or cerebrospinal fluid (CSF) concentration and tissue concentration, and allows for sufficient data for PK modeling. If the PK properties of an ON platform are well understood, then a stand-alone single-dose PK study is not necessary because compounds of the same platform often share similar ADME patterns. The combination of prior knowledge of the platform and PK data from pharmacology and/or exploratory toxicity studies may be sufficient to design the pivotal toxicity studies and FIH studies. An alternative approach to a single-dose PK study is determination of PK/TK on day 1 of a repeat-dose study.

There are advantages and limitations associated with using repeat-dose PK/TK information in lieu of a stand-alone single-dose PK study, as discussed in more detail in Repeat-Dose PK/TK Study section. Study design often depends on the cumulative knowledge of the platform.

In general, the default ROA for animal PK/TK studies is the clinical dose route. When the clinical route is not IV, such as SC, IT, or IVT dosing, a single-dose IV PK study in animals may be needed to characterize the PK properties more completely, including the determination of absolute bioavailability of non-IV dosed ONs. This is often applicable to new platforms or new formulations, and generally for internal decision making, due to the advantage that IV PK provides in characterization of the distribution and elimination phases without being impacted by ongoing absorption. However, for ONs, often bioavailability is not best correlated with PD or toxicity, as such, the requirement of absolute bioavailability determination is not consistent across regulatory agencies. The rapid absorption into the systemic circulation and negligible local metabolism at the site of injection due to a good metabolic stability would argue against the necessity of IV dosing for absolute bioavailability determination.

Similar to that for small molecules, doses for PK studies should consider the pharmacological activity in animal models, interspecies scaling, and anticipated dose range in humans. If no prior PK information on the compound is available to assist with dose selection, *in vitro* potency and PK/PD information of the same class of compounds can be referenced for study design. Prior tolerability information should also be considered, especially for the selection of the top dose as some ONs may present a steep toxicity dose–response.

The species selected for a single-dose PK studies are normally the same species as those used for the pharmacology and toxicity studies, which are generally rodents and monkeys. If adequate information regarding the ON platform exists, a PK study in one species that is more predictive of human PK should suffice. Otherwise, single-dose PK studies in both rodent and monkey may be considered.

Sex differences in PK are sometimes observed in mice, but rarely in rats, monkeys, or humans [18–20]. Therefore, the initial single-dose PK study can be conducted in one sex, for example, males, to simplify the study, and sex-related effects can be evaluated later in repeat-dose toxicity studies.

Additional study elements to consider include study duration, time points, sample matrix, bioanalytical considerations, and compliance with Good Laboratory Practices (GLP). Considerations for these study elements are similar to those for small molecules or biologics. Typically, following systemic administration (SC or IV), ONs are absorbed into the systemic compartment rapidly in both rodents and monkeys. The time to reach maximum observed concentration (T_max_) values in plasma are similar across species (within a couple of hours). Following local administration (eg, IT, ICV, IVT), ONs transfer from the local compartment (CSF and vitreous humor) to the systemic space rapidly. The T_max_ observed for such ROAs is delayed slightly compared with systemic administration. Plasma half-life (t½) for both distribution and elimination varies from days to several weeks depending upon the ON chemistry.

The study duration of a single-dose PK study should be sufficient to understand the PK properties of the molecule, especially as half-lives in plasma may be drastically different for ONs of different design. Ideally, the sampling period should be long enough to cover at least three elimination half-lives. Time points should be selected to characterize peak concentration (C_max_), T_max_, and area under the concentration versus time curve (AUC) adequately; determination of terminal phase parameters such as t_1/2_, clearance, and volume of distribution is optional, but can be critical for building PK/PD or PK models. Dose–exposure relationship should be assessed based on C_max_ or AUC in studies with multiple dose levels. Plasma concentrations during the terminal phase have been found to be proportional to the tissue concentrations [21] and increased plasma trough concentrations can be indicative of antidrug antibodies (ADAs) [22], therefore, it is useful to develop sensitive bioanalytical methods to determine terminal t_1/2_.

The sample matrix is typically plasma (or serum) to assess systemic exposure and may also include local compartment fluid, such as CSF or vitreous humor, to assess local concentrations in cases of local delivery. Sample collection and handling are relatively standard for ONs regardless of the bioanalytical method. The blood samples should be held on ice, centrifuged under refrigeration, and the resultant serum or plasma should be frozen as soon as possible. For mRNA-LNP, a stabilizer needs to be added for mRNA measurement. The ratio of ON concentrations in the primary matrix relative to tissues are often measured; more details on tissue distribution (TD) studies are provided in the Distribution section.

ADAs are usually not measured in a single-dose PK (or PK/PD) study. Stand-alone PK and PK/PD studies not including toxicity endpoints and those that will be superseded by more robust toxicity assessments are usually conducted as non-GLP compliant.

### Repeat-dose PK/TK study

TK characterization is usually recommended for non-GLP toxicity studies and is required for GLP toxicity studies to determine exposure for assessment of safety margin [3]. The general principle is the same for ONs and small molecules/biologics. The main advantages of using repeat-dose PK/TK studies in lieu of a stand-alone single-dose PK study include savings of time, cost, and possibly animals. Additionally, repeat-dose studies enable characterization of TK at steady state and inform on the extent of accumulation. However, collection of TK samples could impact toxicity study operations adversely because of frequent sampling and animal handling, consequently imposing sample volume constraints to avoid confounding toxicity results. Adding sufficient satellite TK animals, composite sampling, or sampling in recovery phase animals can overcome some limitations; however, this may increase cost and add operational burden. Another limitation is that the dose range for toxicity studies is sometimes suprapharmacologic and the linearity may not reflect that of the clinically relevant dose range, which is usually narrower and at the low end of the dose range for toxicity studies.

Considerations for routes of administration, dose, species selection, matrix, and bioanalytical method for single-dose PK studies discussed in Single-Dose PK Study section are also applicable to repeat-dose PK studies.

The considerations for selection of sex, age, duration, time points, GLP compliance, and timing, when designing repeat-dose pharmacology and toxicity studies, are the same for ONs and small molecules/biologics. In toxicity studies, if recovery periods are warranted, their durations should consider half-life of the test article in tissues, as this could influence time to recovery from test article-related adversity. The PK or TK time points should be sufficient to characterize at least AUC, C_max_, and T_max_ following the first and the last dose. In addition, inclusion of a time point just before the next dose is recommended, at least in nonrodents, to determine C_trough_. Dose proportionality and accumulation upon repeated dosing are usually assessed based on AUC and C_max_ from the first and last doses at each dose levels. The study timing is consistent with the GLP toxicity studies recommended by ICH M3(R2) [1] or ICH S9 [10], and are typically assessed before initiation of repeat-dose clinical trials.

#### ADA measurement

The Subcommittee recommends that the approach to assessment of ADA be a risk-based consideration, with principles that are similar to that for biologics per ICH S6(R1) [23] and the FDA Immunogenicity guidance [24], and follow the recommendations of the OSWG ADA Subcommittee [25]. The measurement of ADA in animals should be conducted only if there is evidence of changes in PK, PD, or toxicity that could be explained by ADA. Based on the known difference in immunogenicity response between species, the translational relationship to humans needs to be considered, but these data may aid in overall study interpretation regarding potential human safety.

The timing of assay development and sample collection should be determined based on the observed data, the class of ON being studied (including delivery mechanism), the route and frequency of administration, the urgency of the patient population, etc. Because ADA are typically not evident until at least 1–3 months postdose, the Subcommittee recommends that Sponsors collect samples and be prepared to assess ADA primarily in the chronic toxicity study in monkeys, if warranted. If early rodent toxicity studies reveal changes potentially related to ADA, preparation for ADA assessment in the subsequent chronic toxicity study in rodents may also be warranted.

ADA assay development is a lengthy procedure that is often initiated prospectively in parallel with the chronic study(s) or after the study only if triggered by PK or safety findings. In addition, development of the assay should be preceded by development of a positive control antibody. In general, the FDA recommendations for ADA assay development and validation for therapeutic protein products are applicable to therapeutic ONs [24]. Sponsors should also be aware that the presence of ADA can interfere with detection of the test article by the bioanalytical assay, especially hybridization-based enzyme-linked immunosorbent assays (HELISAs). Therefore, validation of the bioanalytical method may need to assess the effect of ADA (eg, through use of a positive control antibody) or include mitigation to eliminate the effect of ADA on the performance of the assay.

Selection of ADA sample collection time points should consider that the presence of the test article may interfere with the detection of ADA. Samples, either serum or plasma, are usually collected at prestudy, and at time points coincident with PK trough samples during the dosing period and at time points that correspond to other measurements (eg, PK and necropsy) during the recovery period. If sample analysis is warranted (ie, based on observed changes), results should be presented in terms of incidence, onset, and duration similar to that recommended for therapeutic proteins [26].

For ASOs, the most common effect of ADA on PK is typically a time-related increase in trough concentrations, as compared with ADA-negative animals [22]. Similar ADA-mediated effects have also been observed in humans. The effect is dependent upon titer and is seen only at low circulating ON concentrations; thus, C_max_ and AUC are typically not impacted. Assessing the impact of ADA on PK requires a holistic approach of between-subject and within-subject analyses [25] or a simple twofold rule. If trough concentrations are affected by the presence of ADA, determination of the plasma t_1/2_ should be limited to ADA-negative animals only. Similarly, ADA-positive animals should be excluded for PK parameters derived from the terminal elimination rate constant, such as apparent volume of distribution, whereas exposure parameters, such as C_max_ and AUC, can be summarized based on all animals [27,28]. ADAs appear to be less common for siRNAs than for ASOs [25,29]. However, neutralizing anti-PEG antibodies was observed in rats receiving patisiran [30].

#### PK considerations for reproductive and juvenile toxicity studies

The primary purpose of TK characterization in the reproductive toxicity studies, whether fertility, embryo-fetal development, or pre- & post-natal development, is to confirm exposure in the dosed animals, compare with exposure in general toxicity studies to determine if pregnancy alters exposure, and compare with exposure in human to support risk assessment. If such studies are conducted in a species for which TK data are not available (e.g., rabbits), a PK or a dose range-finding toxicity study with TK characterization is recommended to characterize systemic exposure and guide study design prior to initiation of the definitive toxicity study. To facilitate comparison, the TK assessment should employ as many of the same time points as used in the previous studies to the extent practical and feasible. In these studies, it is important to use satellite groups to avoid the potential impact of blood collection on maternal and fetal toxicity. Similarly, the purpose of TK characterization in juvenile toxicity studies is to compare exposure with that in adults. Therefore, if the same ROA is used, use of the same time points is recommended.

### Special considerations

For ONs encapsulated with LNPs, it is advised that, in addition to PK characterization of the ON payload, the PK of any novel formulation components (ie, those that have not been used previously in an approved drug) be characterized following administration of the formulated product [31]. Upon hepatic uptake and ON release subsequent to IV administration, novel LNP components disassociate from the LNP; thereafter, their plasma profiles depend on their equilibrium with the circulatory system and elimination mechanism. Plasma half-life determination of the novel lipid components will provide information on clearance through biodegradation and/or excretion. With regard to ADA, because the ON moiety is encapsulated and, therefore, inaccessible, assessments should focus on antibodies formed against the LNP or LNP component(s) rather than the ON.

For conjugated ONs, it is recommended that the determination of PK of both the conjugated ON and its metabolites (eg, unconjugated ON) be built into the bioanalytical strategy. The kinetics of the conjugate moiety alone, such as GalNAc, are rarely assessed in toxicity studies.

## Distribution

The primary objective of TD studies is to characterize relative ON exposure across organs and tissues as compared with the target organs for toxicity or activity. The purpose of these studies is to understand the tissue PK/TK in relation to PD and/or toxicity. These data may inform the appropriate dosing frequency and/or PK/PD in nonclinical and clinical programs.

### *In vitro* plasma protein binding

Plasma protein binding (PPB) appears to play a very different role for ONs than for small molecule drugs. It is well accepted that PPB tends to decrease glomerular filtration and renal excretion and, thus, increase a drug's time in circulation; this is true for ONs [32] as well as small molecules. For the latter, PPB decreases availability for cellular penetration and, therefore, pharmacological activity. In contrast, PPB of ONs may actually be important for cellular uptake through endocytosis and can probably improve pharmacological activity [33]. Transiently increased activated partial thromboplastin time and complement activation have been attributed to high PPB [34], although these effects are rarely observed with siRNAs [35]; see also Janas *et al.* [36] for discussion. Overall, the binding sites for ONs (hydrophilic) differ from those for small molecule drugs (hydrophobic); binding affinity is generally low and binding is transient [37–39]. Importantly, PPB of ONs is generally similar across species. For a PS ASO, ranges were comparable for rat, monkey, and human, but slightly lower for mouse [38]. No species difference was apparent for an siRNA [40].

Protein binding of ONs is a function primarily of the chemical modifications, with other attributes, including sequence, being less of a factor. PS modifications increase PPB, whereas 2′-MOE and 2′-cEt modifications result in lower binding [41] and 2′F has no apparent effect [40]. Thus, ASOs with high PS or high locked nucleic acid content tend to have high (>95%) PPB [21] as can siRNAs with high PS content [40], although the basis of binding appears to be intrinsically different for the two classes [42]. In general, single-stranded ONs demonstrate higher binding than duplexes; strand flexibility also affects binding, with polyA showing 100-fold lower affinity than polyT [41]. Although GalNAc conjugation tends to decrease binding [40], PS modifications remain the primary factor; a GalNAc-conjugated siRNA with minimal PS content can exhibit substantially lower PPB (54%) than a GalNAc-conjugated fully PS ASO of the identical sequence (99%) [42]. As further contrast, eteplirsen (PMO) containing no PS modifications, exhibited even lower PPB (0.2%–25.4%) due to its uncharged backbone [43].

ON length is comparatively unimportant; for one 20-nucleotide-long ASO, the extent of binding was relatively constant among most metabolites and did not decrease until the nucleotide chain length was ≤10 bases [38]. Sequence may also contribute but appears to be substantially less important than the chemistry, particularly for PS ONs [38,40,41]. A recent comprehensive review provides specific information of the effects of and factors affecting PPB of PS ASOs [44].

Because binding is typically similar for compounds within a platform, the Subcommittee advocates use of a platform-based approach. For ONs of well-understood chemistry, a limited approach to PPB evaluations, such as described below, or a paper argument may be sufficient; this is in line with the recommendations of Humphreys *et al.* [45]. Similarly, binding data may not be warranted for naked ONs where lack of protein binding is well established (eg, PMOs), or for formulated Ons, or in cases where a variety of methods have failed.

If conducted, the evaluation should be sufficient to determine the extent of binding and to assess any species-related differences in PPB. At least semiquantitative determination is recommended and should include plasma from humans and the toxicology/pharmacology species used in the nonclinical program. Concentrations should range from clinically relevant to C_max_ in the nonclinical toxicity studies (generally several orders of magnitude). At least three concentrations should be tested. However, determination of saturation is not required. If nonclinical species data demonstrate notable differences in kinetics or toxicity, further characterization of PPB may aid in the elucidation of these differences.

The matrix in which PPB is characterized can typically be limited to plasma for ONs that are administered parenterally. However, for test articles administered locally (eg, IT, ICV, or IVT), testing should probably include the local matrix (eg, CSF and vitreous humor), as protein binding in CSF or vitreous humor can dictate half-life of the ON in these matrices and, therefore, would impact the distribution in target tissues (brain or eye). A paper argument to describe the PPB characteristics can be made based on the understanding of the protein content in the matrix [38].

Gel shift or electrophoretic mobility shift assay (EMSA) is a commonly used method for semiquantitative determination of PPB *in vitro*. For quantitative determination, ultracentrifugation, ultrafiltration, and equilibrium dialysis can be used. Nonspecific binding observed in ultrafiltration methods can often be minimized by use of detergents and/or pretreatment of the filter with a high concentration of a “scramble” or nonspecific oligo that has a similar chemistry to the test compound. New methods (eg, bead-based) may need to be developed for ONs with new chemistries. Regardless of the partitioning method selected, a highly sensitive bioanalytical method is needed to quantify the low concentrations of unbound ON. Radiolabel methods may be feasible, but purity of the tracer should be high enough for such determinations.

Although ICH M3(R2) [2] indicates that *in vitro* PPB data should be available before initiation of FIH trials, this timing may not be as important for ONs as for small molecules given the limited species-related differences in PPB of ONs as discussed above. Animal toxicity or pharmacology studies are highly likely to be predictive of any PPB-related effects in humans; therefore, the risk of initiating clinical trials without prior PPB data appears to be low. Nonetheless, protein binding data may have value in development with regard to understanding of any safety findings from either the parent ON or metabolites. Furthermore, for certain nonclinical studies, such as *in vitro* DDI investigations (see *In Vitro* PK Drug Interactions section), test concentrations do not need to be adjusted; this is in contrast to studies for small molecules for which this adjustment is needed to account for protein binding and to ensure exposure to free (unbound) drug. Last, PPB is usually similar across species; however, if PPB differs significantly between nonclinical species and humans, the difference should be taken into account in potential human systemic exposure and risk assessment.

Therefore, Sponsors should plan to generate PPB data either before investigational new drug (IND) application or in parallel with Phase 1/2 studies, if justified, as discussed above.

### *In vivo* TD

*In vivo* TD is expected to be determined before large-scale clinical trials (eg, Phase 3) or registration but is typically assessed much earlier in development for ONs than for small molecules or biologics. Because chemistry can influence uptake, distribution is often assessed during candidate selection to optimize delivery. Distribution is fairly well documented for ONs with standard chemistries (eg, GalNAc, PS, 2′-MOE, etc.), but studies may have value for ONs with novel chemistry and/or targeting ligands. For conjugated and LNP-formulated ONs, distribution of the ON as well as any novel component(s) may need to be determined after administration of the drug product (see Special Considerations section). The extent of the assessment depends on the type of chemistry, ROA, distribution of target gene, predicted organs of accumulation (eg, liver, kidney, site of administration), and stage of development. Regardless, TD data can be useful for resolving residual uncertainties regarding the relationship between a toxicity finding and tissue exposure. TD studies also provide information about tissue residence time, which could be relevant to PD (eg, dosing interval) and/or toxicity (eg, reversibility).

The design considerations for TD studies of ONs are similar to those for small molecule drugs. A typical design would entail a single administration at a clinically relevant dose (eg, in the range of pharmacologic activity). Dose levels administered in toxicity studies may be saturating and result in distribution that is not comparable to what would occur clinically; however, assessment at such dose levels may have utility in explaining differences in toxicity between a clinically relevant dose and a toxic dose level. Studies are usually conducted in a single species (ie, rodents) and a single sex. The use of an alternative species or an animal model may be warranted based on the ROA or clinical indication. Typically, 4–7 time points are assessed to determine tissue half-life. Time points are often selected to cover plasma T_max_, tissue T_max_, and ideally approximately four to five tissue half-lives; therefore, studies can be quite long in duration, particularly for ONs that are designed to be stable. These investigations are often conducted as stand-alone studies rather than incorporated into toxicity studies.

To assess accumulation, distribution can be determined after single- and repeat-dose administration or estimated based on tissue half-life from single-dose studies and the intended dosing frequency. That is, if well characterized, single-dose tissue TK data can be used to simulate repeat-dose tissue TK with reasonable accuracy. Tissue collection at multiple time points, such as at the end of recovery periods in toxicity studies, may provide useful information. Specific tissues for collection will likely be based on the results of PD and/or toxicity studies. Assessment of exposure levels and or target engagement (PD) will depend on the individual program and stage of development. The steady-state plasma and tissue TK data from monkey and other animal models (as appropriate) can be used to estimate tissue concentrations in humans with proper interspecies scaling [21,37,46–49]. Some Sponsors have opted to conduct these multidose TD studies to support FIH trials; however, the applicable guidance is unclear about the required timing for such evaluations.

#### Systemic administration (IV and SC)

A broad TD assessment may be conducted and would include organs of high uptake, such as kidney and liver, in addition to the target organ(s) related to the therapeutic indication. Determining distribution in organs that specifically express the pharmacological target might also be warranted, if that is not the primary organ related to the indication. Additional tissue evaluation in subsequent studies may be warranted based on the results of pathological findings in toxicity studies.

#### Local administration

Following local administration, distribution should be characterized for the various matrices associated with the site of administration (eg, vitreous humor and retinal layers of eye following IVT administration or CSF, brain, and spinal cord sections following IT or ICV administration) where possible; care should be taken to avoid blood contamination during dissection. Information on local versus systemic exposure can be used to select appropriate dose levels for systemic toxicity studies. Egress of the test article from the local site or compartment into the systemic circulation and subsequent systemic exposure should be characterized in conjunction with the local exposure. Slow egress or depot effects should be considered carefully with regard to timing of PK sampling. Prolonged (although likely low) systemic exposure is likely after local administration [37].

#### Bioanalytical methodology and tissue collection

In general, tissue concentration determination utilizes non-GLP methods. Methods are typically “qualified” rather than “validated.” Tissue collection methods should consider homogeneity of ON concentrations within the organ. For instance, concentrations vary considerably between kidney cortex versus medulla or different anatomical regions of the brain; therefore, care should be taken to ensure consistent sample collection among animals to minimize variability. In contrast, concentrations in liver are relatively constant among the lobes; therefore, consistency during tissue collection is less critical. Assessment of exposure in different anatomical regions of an organ (suborgan and/or cellular distribution) can be conducted for research purposes to understand the pharmacology or specific toxicity; there is no regulatory requirement and assessment in one species (eg, monkey) and not the other (eg, mouse) can confound cross-species comparisons. Nonquantitative techniques such as *in situ* hybridization (ISH) could aid in determining detailed tissue and cellular distribution.

It should be recognized that assessment of TD through unlabeled methods can result in an incomplete picture of distribution due to collection of limited number of tissues and/or the inability to detect metabolites; however, if sufficient distribution data exist for the platform, a cold method should suffice.

Limited TD assessments are typically included in EFD studies [50]. Concentrations in maternal tissue and/or plasma are measured in satellite animals and are used to confirm exposure and allow for comparison to exposure in the general toxicity studies. If possible, sample collection time points should be the same as those used in previous studies to facilitate comparison. Assessment of exposure in the fetuses and pups can be limited to the major organ of uptake (eg, liver) to determine whether the ON crossed the placental barrier and/or was bioavailable through the mother's milk; assessment of exposure in the placenta is not necessary to achieve this goal.

#### Radiolabel and other imaging TD studies (see also discussion re: excretion)

The use of radiolabeled test article in TD studies is not necessarily required by regulatory agencies because the TD of ONs is generally considered well understood and consistent across species. If sensitive bioanalytical methods of detection with high dynamic range are available that can quantify ON concentrations in various tissues, then radiolabel studies may be of limited additional value. If radiolabeled TD studies are conducted, the location of the radiolabel within the ON should be selected with consideration for metabolism and potential detection of shortmers or reincorporated monomers. The stability of radiolabeled ONs does not appear to be problematic unless specific activity is too high. To ensure stability (and because of expense), ONs are typically labeled on only 1–2 bases per molecule. Although assessment of TD through radiolabel provides certain advantages over cold methods, this approach is more expensive and time consuming.

If radiolabeled TD studies are conducted, the typical methodology is quantitative whole-body autoradiography (QWBA) in rats. However, regardless of the methodology, such studies have their limitations because the read-out (ie, radioactivity) does not distinguish between active parent (or active metabolites) and inactive metabolites. Therefore, an appropriate “cold” method such as liquid chromatography–mass spectrometry (LC-MS) may be needed in conjunction with the radiolabel to distinguish parent from metabolite. Metabolite identification (ID) conducted on tissue homogenates at a few selected time points (eg, peak and trough) can provide information relevant to interpretation of the studies.

Other imaging approaches to consider for investigating the distribution of ONs in tissues, even at the cellular level, include fluorescent *in situ* hybridization (FISH) using a fluorescent-labeled probe complementary to the ON of interest and immunohistochemistry (IHC) using an antibody to the ON. The distribution of the ON in histological sections using FISH can be visualized by confocal microscopy, whereas those stained by IHC are examined by light microscopy. Both techniques can be used only in a qualitative and not in a quantitative manner. Metabolites of the ON might also hybridize with the FISH probe or bind to the antibody, which may influence interpretation of the results; this possibility can be checked during the method development phase. Despite a few downsides, such assays can help the Sponsor evaluate cell-specific uptake of the ON, which is essential for developing treatments for diseases in which the pathology originates from a specific cell type (eg, retinal ganglion cells in retina or neurons in brain). Such information is not available from quantitative assays such as LC-MS, HELISA, or imaging methods such as QWBA.

Both albino and pigmented animals are typically included in radiolabeled studies of small molecules to allow for determination of binding to melanin. However, this determination is rarely considered necessary for systemically administered ONs; phosphodiester ONs have been shown not to bind to melanin [51]. For ONs administered intravitreally, the use of pigmented animals may be requested for determination of distribution [52].

### Special considerations

#### ONs with targeted delivery

For conjugated ONs, the TD of both the conjugated ON and its metabolites (eg, unconjugated ON or the parent drug ON) need to be assessed. The fate of the conjugate moiety (eg, GalNAc) is often assessed during metabolism/distribution aspects of repeat-dose toxicity study(s). Therefore, analysis is typically cold (nonradiolabeled) and may be limited to the target organ of PD, liver, kidney, and other relevant organs at limited time points. The linker is often designed to be labile, that is, to release the conjugate immediately upon cellular uptake of the conjugated ON by receptor-mediated endocytosis. Indeed, in a pilot ADME study of a GalNAc-conjugate in which the linker was radiolabeled to understand the metabolic profile of the conjugate, the linker was removed rapidly [53]. TD data should be supplemented by information on the tissue expression of the receptor (and/or the PD target) across species. This information should be provided early in clinical development (eg, IND) in the absence of comprehensive TD data, and then later correlated with TD data once available.

For LNP-formulated ONs, distribution of each novel component should be determined as appropriate.

- For LNP-formulated ONs (siRNAs or mRNAs), distribution of the ON payload is determined in the same manner as for an unconjugated ON, as discussed above. The biodistribution and persistence of the ON should be determined in all relevant organs and tissues, whether intended target or not. The study should mimic the clinical use with regard to dosing and ROA, but the ROA that results in maximum systemic exposure of the ON (eg, IV administration) may be included. The sampling period should allow for determination of the maximum concentration of the administered material and continue until concentrations are below the limit of quantification or a plateau phase is reached.
- Distribution of any novel LNP components should also be assessed and the tissue half-life determined. Although TD of the LNP is often conducted early in development through fluorescence using green fluorescent protein or luciferase-encoding RNA as surrogate payloads, such studies provide insufficient information on the novel component(s). Assessments using bioanalytical techniques specific to the novel component(s) should be conducted before large-scale clinical trials (eg, Phase 3). However, the timing will be dictated by the findings in the toxicity studies. If observed toxicity cannot be ascribed to the ON (eg, exaggerated pharmacology, off-target RNA-directed effects, or known class-related effects), then the potential for the novel LNP component(s) to accumulate and cause toxicity (eg, local irritation, complement activation, systemic inflammatory response, etc.) should be assessed earlier in development.
- For LNP-formulated mRNA therapeutics, the expressed protein should be determined in the target tissue or, if the protein is secreted, in plasma.

A platform-based approach, as described in the 2020 FDA guidance [54], should be an option for TD studies of conjugates and LNPs. Such an approach was used successfully in the development of LNP-formulated mRNA vaccines to prevent COVID-19.

## Metabolism

Due to the consistency of ON metabolism across species, the potential for metabolites that are either unique to humans or present at disproportionately higher levels in humans than in any of the animal test species (disproportionate human metabolites) is substantially lower than for small molecules. The metabolic pathway is known (cleavage by exo- and/or endonucleases) and no species-, sex-, or age-related differences in these enzymes in mammals have been reported to date. Metabolism through other pathways (eg, deamination) appears to be rare.

Because the metabolites are the result of cleavage rather than biotransformation through Phase 1 and 2 enzymes, their toxicity is considered similar to that of the parent ON [55]. On the other hand, large metabolites of ONs (ie, those resulting from the cleavage of a small number of nucleotides from the ends) are likely to be active; as such, quantitation of the parent alone may lead to underestimation of PD activity. In addition, smaller metabolites (shorter sequences) might hybridize with off-target sequences in the genome, resulting in undesired effects. Furthermore, assessment of novel formulation components and novel conjugates/linkers must also be considered. Overall, metabolite studies are important for ON drug development. The Subcommittee separates the nonclinical metabolite investigations of ONs into two categories as follows:

- Informational investigations: These include *in silico* searches to minimize potential for off-target effects due to any chain-shortened metabolites as well as *in vitro* metabolism studies to facilitate selection of new chemistries or lead candidates. It may also include *in vitro* or *in vivo* metabolism data for risk assessment or method development. For instance, tissue metabolite data in animals could be obtained for target engagement purposes; similarly, *in vitro*, tissue, and/or urine metabolites could be determined to help anticipate potential human metabolites. Such studies should be conducted at the discretion of the Sponsor rather than as regulatory requirements.
- Safety-related investigations: Such studies pertain to the identification of major human metabolites and comparison of exposure in humans versus the nonclinical toxicity studies as described in the FDA MIST guidance [5]; the only relevant matrix is plasma. For any unique or disproportionate human metabolites, the potential for pharmacological activity or adverse effects related to off-target hybridization-dependent toxicity should be assessed [56]. Because this evaluation is predicated on the availability of human samples at steady state, it should be conducted concurrent with the Phase 1 multiple dose or Phase 2 clinical trials.

Regardless of the category, each type of study is described in more detail below. However, it is important to recognize the differences in metabolism among different types of ONs. The recommendations above should be assessed on a case-by-case basis with scientific rationale. For example, metabolism of ONs using novel chemistries and/or novel components should probably be investigated earlier and more comprehensively than those using only established chemistries. In contrast, metabolism investigations may not be applicable directly to mRNA-based therapeutics if their degradation results in only naturally occurring nucleosides. Similarly, metabolism data are not needed for an animal-active analog.

### *In vitro* metabolic stability

*In vitro* metabolic stability assays for ONs play an important role at the discovery stage to evaluate new chemistries and/or to select compounds with improved stability. The assays are typically conducted with qualitative assessment of the remaining intact full-length ONs at specified time points [57]. Comparison across species, if conducted, may identify differences in the rate of metabolism but rarely identifies different metabolites. Selection of the test system(s) should be based on the study objective. A suitable and optimized *in vitro* test system would help predict and evaluate to gain a better understanding of sequence and chemistry alterations that may influence metabolism *in vivo*. For instance, purified nucleases or liver lysates/homogenates might help identify “weak points” in a new chemistry or a particular sequence. Plasma and/or target tissue cells or homogenate at physiological conditions would be more appropriate for prediction of *in vivo* stability. Because the *in vitro* study duration is much shorter (1–5 days) than the half-life in tissues, the results help to rank the ONs in terms of stability but do not predict the *in vivo* half-life.

For ONs that are administered locally (eg, IT), the local matrix (eg, CSF and/or brain homogenates) should also be considered. Although species differences in metabolism are not typically observed for ONs, human-based *in vitro* assays may provide a more precise prediction of human outcomes. Nonetheless, some Sponsors also investigate *in vitro* stability in the species used for pharmacology and toxicity studies because stability (typically the rate of metabolism) can differ across species. Because one established test system does not fit all situations, the Sponsor must find the right test system to address the specific metabolism-related questions.

### *In vitro* metabolite profiling/identification

The objective of *in vitro* metabolite profiling/identification of small molecule drugs is to determine whether metabolite profiles are similar across the species (ie, the potential for unique human metabolites). ICH M3(R2) [2] recommends that *in vitro* metabolite data (eg, profiling/identification) be conducted before IND. In addition, the MIST Guidance [5] recommends that cross-species comparisons of *in vitro* metabolites occur early in the drug development process to enable more accurate predictions of a drug candidate's safety performance during clinical trials.

For ONs, the data are considered of minimal value for human safety due to the consistency of metabolic pathways across species. Therefore, the Subcommittee feels that they should not be considered essential. In cases where the Sponsor elected not to provide *in vitro* metabolite data in the IND, the absence of data did not lead to clinical hold. Also, some Sponsors chose to meet this “requirement” with *in vivo* metabolism data from the toxicity species, which are of greater value than *in vitro* data, including from human matrices. It is also possible to address the question through an article discussion mentioning the metabolic pathway [ie, nuclease-mediated, not cytochrome (CYP) 450-mediated for naked ONs], stating that no species-related difference in metabolism are observed for ONs, and that metabolism (and metabolites) has no impact on safety relative to parent. This approach should be applicable to all ONs, particularly those with well-established chemistries for which the metabolite pathways and profiles have been well characterized.

On the other hand, some Sponsors find that *in vitro* studies provide a preliminary assessment of likely *in vivo* metabolites; such information has utility in setting up targeted methodologies for analysis of *in vivo* samples. If *in vitro* studies are conducted, they are typically conducted with rodent, nonrodent, and human matrices. Hepatocyte cultures are a good *in vitro* model because they provide the closest approximation of uptake into specific compartments of hepatocytes *in vivo*, whereas liver S9 or tissue homogenates can result in exposure to nonrelevant compartments and, thus, overexposure to nucleases relative to *in vivo* systems.

### *In vivo* metabolism

The primary objectives of *in vivo* metabolism studies are to identify metabolites and ensure safety coverage for those found in human plasma. Fortunately, the metabolic pathway of ONs is generally predictable, which facilitates identification of metabolites. An additional objective is to identify whether any impurities in the drug substance are also *in vivo* metabolites; identification of such impurities has utility because they are generally qualified during the toxicity evaluations of the drug substance as per ICH Q3B(R2) guidance [58].

ICH M3(R2) [2] and ICH S9 [10] recommend determination of *in vivo* metabolites before large or long-term clinical trials (eg, Phase 3). The Subcommittee is in general agreement with this timing because the data have little to no value until clinical samples are available for comparison (eg, end of Phase 1 or concurrent with Phase 2). Deferring until before Phase 3 may be appropriate especially for well-established platforms. Nonetheless, some Sponsors may elect to conduct method development, metabolite profiling, and/or metabolite identification earlier.

Species and sexes used for *in vivo* metabolism studies are usually defined by the toxicity studies. Unless sex-related differences in exposure to parent ON were evident, metabolite investigations can be limited to one sex.

The primary matrices for metabolite evaluation are plasma and urine due to ease of collection. Metabolites in plasma during the elimination phase are considered representative of those in tissues. Urine is not a good substitute for plasma due to further metabolism of ONs in the kidney and because certain metabolites in plasma may not be present in urine. Secondary matrices (tissues) could be considered to understand the exposure related to PD activity and toxicity. Choice of matrices should be based on the major organs of delivery or tissues that express the PD target, such as liver, kidney, and the site of administration. Tissue selection is driven largely by the Sponsor's desire to understand metabolism, rather than any specific regulatory requirement. For instance, for an established platform, a Sponsor may elect to limit investigations of metabolites in the pharmacological target tissue at clinically relevant doses. Regardless, tissues assessed are typically those expressing the PD target, those with presumed high uptake and/or long retention times, and those with test article-related toxicity.

For ONs employing organ-specific delivery, metabolite exposure in the target organ is the more accurate assessment of the exposure margin between human and animals used in the toxicity studies. However, most of these organs are not accessible for measuring metabolite exposure in the clinic. Although the PK profiles of ONs often differ between plasma and tissues, the concentration in tissues is proportional to that in plasma in the terminal phase, as mentioned in Single-Dose PK Study and *In Vivo* TD sections. Therefore, plasma exposure should still provide meaningful and reasonable assessments on safety coverage of metabolites.

Analytical methods used in metabolite evaluation are generally limited to LC-MS for all matrices due to its ability to identify the exact ON sequence. Other techniques such as hybridization-based ion exchange chromatography could be used for quantitation of metabolites as they can distinguish between N-x metabolites, although not as well as LC-MS-based approaches. HELISAs are not recommended unless they can distinguish between parent and metabolites [59,60].

Nonradiolabeled metabolite analyses are generally incorporated into toxicity studies. The best approach would be to conduct the study at both the no-observed-adverse-effect level (NOAEL) and one other dose level to establish linearity for extrapolation. Radiolabeled studies are not typically used for metabolite evaluation of ONs, unless conducted in conjunction with a more specific analytical method such as mentioned above.

### Safety testing of metabolites

The FDA MIST guidance [5] describes the recommended approach to identifying unique or disproportionate human metabolites and determining whether such metabolites should be considered for safety assessment. Human metabolites that can raise a safety concern are those present at >10% of total parent drug-related exposure at steady state [2,5]. As noted, the guidance does not necessarily apply to cancer therapies; the safety evaluation of disproportionate human metabolites in animals is not generally warranted for advanced cancer indications [10].

Although the FDA MIST guidance was developed based on small molecules, the same principles should apply to ONs even though the probability of such metabolites occurring is substantially lower. If the comparison identifies any unique or disproportionate human metabolites (ie, those that were not qualified by the nonclinical safety evaluation of the full-length parent compound), they should be reviewed to determine whether further nonclinical studies are warranted to assess their toxicity. Given that ONs are not metabolized through Phase 1 metabolic pathways, there is virtually no concern about chemically reactive metabolites [21]. Metabolism is mediated by nucleases and typically results in shorter chains with sequence and chemistry derived from the full-length parent compound. Thus, metabolites can be pharmacologically active and/or have the potential for toxicity through off-target binding.

In rare instances, metabolism could also result in a conversion of nucleotides, resulting in a metabolite with an altered sequence; for instance, a metabolite resulting from deamination of the 3′ terminal 2′-O-methyl-adenosine to form 2′-O-methyl-inosine (A-to-I editing) was identified in monkey liver [61]. An analytical method such as liquid chromatography–high-resolution mass spectrometry (LC-HRMS) is required to identify such metabolites.

The recommended approaches to metabolite assessment are described below.

It should be noted that, if any major metabolites are also impurities in the drug substance or drug product, the safety evaluation of these major metabolites also serves to qualify these impurities [10,55].

#### Active metabolites

In addition to the MIST-based safety considerations described above, it is also important to measure the pharmacological activity of any major metabolite. This is typically determined *in vitro* by the same method used for the parent ON and should be quantitated relative to parent activity.

Metabolites resulting from removal of terminal nucleotides (eg, N-1, N-2, etc.) are often active, although activity decreases with decreasing length. It is unknown if deamination metabolites are active. Greater exposure to active metabolites in humans could lead to greater pharmacological activity than tested in animals; therefore, understanding the contribution of the metabolites to the PK/PD relationship is important. Disproportionate exposure to active metabolites of most ON classes, such as ASOs and siRNAs that cause gene knockdown or silencing are highly unlikely to cause adverse effects if the toxicity studies were conducted at dose levels associated with maximal activity. On the other hand, exposure to active metabolites of other classes, such as immunostimulatory ONs, aptamers, microRNA (miR) mimics that are agonists, mimics, or cause upregulation could lead to exaggerated pharmacology. In either case, if the extent of activity was not assessed during the nonclinical toxicity studies in at least one pharmacologically responsive species, then the potential for such exaggerated activity to be associated with adverse effects should be considered.

Additional nonclinical studies of the active metabolite, as described in the MIST guidance, might be warranted, but must be conducted in a species in which it is active.

#### Off-target effects of metabolites

One concern about ONs is the potential to cause unintended effects through off-target binding. This potential is typically assessed through a sequence comparison to the human genome, as described in [62] as well as the FDA guidance on developing drug for the treatment of chronic hepatitis B virus [63]. Although this assessment will likely cover many potential metabolites, it is possible for a metabolite to have different hybridization-dependent off-target effects than the parent ON. Therefore, the analysis should also be conducted for any unique or disproportionate human metabolites that were not covered by the previously completed bioinformatics assessment.

### Special considerations for novel conjugates and novel formulations

Most conjugated ONs use a naturally occurring ligand, such as GalNAc, peptide, or lipid, as a delivery moiety to deliver the ON to the target cell. After uptake of the conjugated ON into the target tissue, the targeting ligand is cleaved, most likely by lysosomal or cytosolic enzymes, to release the naked/unconjugated ON in the cell. Thus, systemic exposure is almost entirely to the conjugated ON and, assuming rapid and near-complete metabolism, the target cell exposure is almost entirely to the ON itself. A small percentage of the naked/unconjugated ON could be released from the target tissue and, thus, contribute to the systemic exposure and the terminal phase of the PK profile. Therefore, total plasma exposure, as well as the relative contributions of the parent and each metabolite, should be assessed in the toxicity studies. This can be addressed in either the toxicity studies or separate studies and may require development of new bioanalytical methods as some, such as hybridization-based methods, do not discriminate between the two forms for the ON. Many Sponsors also assess the metabolism of the conjugated ON in tissues.

A question has been raised as to whether conjugated ONs should be classified as prodrugs. Clearly, some, such as those using protective groups along the backbone [64], require cleavage of the conjugated moiety(s) for activity and, therefore, meet the definition. However, others may not, depending on the mechanism of action. Regardless, it is the Sponsor's responsibility to demonstrate the safety of the released moiety. In addition, comparison of exposure to metabolites across species is required to determine whether the MIST guidance applies. In many cases, the naked/unconjugated ON and/or shortmers are neither unique nor disproportionate human metabolites; the high systemic exposure to the metabolite(s) achieved in nonclinical toxicity studies often (eg, givosiran) qualifies them, and the MIST guidance does not apply.

The safety evaluation of any metabolites from the delivery components such as LNP or ligands/linkers in conjugates should also be considered, as described by Andersson and den Besten [65]. Typically, this investigation should be limited to novel components that are not naturally occurring or have not been used in other approved therapies. The timing for these studies should be aligned with the metabolism studies for the ONs. For example, patisiran employs an LNP formulation that contains 4 lipid components. For two of these, cholesterol and DSPC (1,2-Distearoyl-SN-Glycero-3-Phosphocholine), assessment of metabolism was not warranted because they have been used in previously approved drugs. In contrast, the other two, DLin-MC3-DMA ([6Z, 9Z, 28Z, 31Z]-heptatriaconta-6, 9, 28, 31-tetraen-19-yl-4-[dimethylamino] butanoate) and PEG_2000_-C-DMG [3-[3-(2-methoxyethoxy)propylcarbamoyloxy]-2-tetradecanoyloxypropyl] tetradecanoate, were novel and, therefore, their metabolite profiles were studied [30,66].

Since the metabolism of the novel excipients is independent of the sequences of the ONs, the Subcommittee recommends a platform-based approach; metabolism work on the delivery components for subsequent compounds of the same platform is not warranted.

## Excretion

Nonclinical excretion studies are conducted to help guide the design of clinical studies for determination of the metabolic fate and elimination routes of the parent drug and its metabolites, which will help inform a decision about the necessity of clinical studies in patients with renal and/or hepatic impairment. However, due to the long tissue residence time for many ONs, it may not be ethical to conduct a radiolabeled mass balance study in humans. In these cases, nonclinical excretion studies may be used to substitute for a human mass balance study. Generally speaking, excretion pathways tend to be less complicated for ONs than for small molecule drugs as the route of excretion is often similar for a class of ONs. Most ONs are eliminated mainly through metabolism in tissues, with typically only a small fraction excreted as unchanged parent. The extent of renal excretion is dependent on the extent of PPB. Safety concerns related to persistence (eg, of modified nucleotides) within the body are more appropriately addressed through distribution studies in conjunction with toxicity studies.

The Subcommittee recommends determination of excretion before large or long-term (eg, Phase 3) clinical trials, as indicated in ICH M3(R2) [2]. If possible, the Subcommittee advocates that a platform-based approach be used to limit the extent of excretion studies warranted for new compounds of the platform. That is, if initial studies confirm results similar to those obtained with prior compounds of the platform, then no in-depth characterization of excretion should be needed. In some cases, a paper argument may suffice in lieu of experimental data, as was done for nusinersen [67].

### Study design considerations

Excretion may be evaluated after a single dose. This can be accomplished in a stand-alone PK study (radiolabeled or nonradiolabeled) or by incorporation into a toxicity study. In either case, the study can be conducted in a single species. Regardless of the species selected, metabolites should be evaluated. The study should include a clinically relevant dose level as well as a high dose level. For some ONs, urinary metabolites change over time as metabolites are slowly released from tissues back into plasma; therefore, the metabolite profile after repeated administration may differ from that after single administration. Based on considerations described in the Metabolism section (eg, activity and safety profile of the *in vivo* metabolites and their relative concentrations), the Sponsor may decide to evaluate metabolites in excreta after repeated dosing, if deemed more relevant, based on the nature of the metabolites.

The goal of excretion characterization should be to determine the percent of administered dose recovered in the excreta. In general, the analyte should be total active compounds (parent plus active metabolites if any) rather than simply the parent drug. Determination of excretion is typically limited to urine. Urine should be collected on ice to minimize metabolism by bacteria from fecal contamination; no chemical stabilization is necessary, but some Sponsors add surfactant or albumin to reduce nonspecific binding. If urine is determined not to be the primary route of elimination, additional matrices (eg, bile) should be considered. In addition, assessment of excretion in feces or expired air is needed as follow-up investigation in the event of toxicity in the gastrointestinal tract or lungs, respectively, and should be considered for ONs administered orally or through inhalation, respectively. If collected, feces should be homogenized with buffer to maintain the pH.

Collection of urine over 24 h (in two to three intervals) postdose is generally considered sufficient for qualitative comparison of metabolite excretion across species. Quantitative assessment of excretion of the parent and metabolites in a relevant animal species, which requires determination of urine volume over one or two 24-h interval, can be used to determine whether urine is the primary route of excretion [68].

### Radiolabel studies

Radiolabel mass balance studies in animals are not a regulatory requirement but have been conducted for the majority of the ONs approved to date. The Subcommittee was somewhat ambivalent with regard to such studies. It was agreed that mass balance may be of less value for ONs with standard chemistries; distribution and excretion are fairly well documented and, therefore, are of limited concern. For these reasons, radiolabel studies may be supplemented by more specific methods of detection.

On the other hand, radiolabel mass balance studies may have value under the following circumstances:

- For locally administered ONs when other methods of analysis are not sufficiently sensitive,
- If fecal excretion is suspected to be a major route of elimination,
- To provide in-depth information on new platforms or novel chemistries (in the ON, formulation, and/or conjugate),
- If needed to provide dosimetry information for clinical mass balance study,
- When the residence time of parent ON or metabolites in the tissues of animals is sufficiently long to preclude a radiolabeled PK study in humans. In this case, a radiolabeled mass balance study in a single animal species combined with a nonradiolabeled clinical excretion study may provide sufficient data.

The decision regarding whether to conduct radiolabel studies is left to the Sponsor. If a nonclinical mass balance study is not conducted, nonradiolabeled assessment of urinary excretion of parent and metabolites should suffice for established platforms. If such a study is conducted, the location of the label within the molecule should be selected carefully as discussed in the Radiolabel and Other Imaging TD Studies section. To minimize potential for misleading or confusing results, the radiolabel should be placed at a nucleolytic-stable position of the molecule. The study design can be fairly limited and is comparable to that for small molecules. A single species is sufficient so long as it has a plasma metabolite profile similar to human; rodent (rat) is usually selected. Similarly, a single sex (usually males) is adequate unless data suggest sex-related differences in plasma PK exist. A single dose at a single clinically relevant dose level is acceptable; however, if TD is assessed in the same study, a high-dose level may also be included to improve detection of the test article in nontarget tissues. Typically, radiolabeled methods (eg, liquid scintillation counting, LSC) are used for excreta.

Depending on the location of the radiolabel, an assessment has to be made to what degree LSC can distinguish between parent and metabolite. Alternatively, a bioanalytical method that is more sensitive and/or accurate can be used. Regardless, the analytical method must be justified but can be qualified rather than validated, and the study conducted as non-GLP. Experience among the Committee members varied regarding the need for quantitative assessment (eg, over 2 days postdose) or for an acceptable percent recovery.

### Other considerations

Assessment of the impact of renal and/or hepatic impairment on PK may be warranted due to the contribution of renal or hepatic clearance (of parent and metabolites) to total clearance. However, the Subcommittee does not recommend a nonclinical approach to assessment. Although ON distribution and excretion has been assessed in rats with experimentally induced renal injury [69], the Subcommittee felt that this was not an appropriate approach or a sufficient substitute for clinical data. Information on the recommended clinical approach has been provided by both the EMA and the FDA [8,70,71].

Determination of excretion in milk is generally not considered warranted, as stated in ICH S5(R3) [72]. For those approved ONs for which excretion in milk was determined, levels were either low or undetectable [30,73,74]. Nonetheless, the Subcommittee recommends that Sponsors consider the level and duration of systemic exposure, the oral bioavailability, and whether the patient population includes nursing mothers. If determination of concentration in milk is warranted, an appropriate bioanalytical assay will be needed. Additional PK information that may be of value in assessing the risk to breastfed infants includes stability in simulated gastric fluid and liver concentration in pups nursed by dosed dams.

Special considerations for different types of ONs are similar to those discussed in the Absorption, Distribution, and Metabolism sections. Excretion of novel components in formulated or conjugated ONs should be determined after administration of the drug product rather than administration of the novel component alone; in these cases, the results can be applied to a platform approach for subsequent compounds using the same formulation or conjugate. For mRNA therapeutics, *in vivo* studies to assess mRNA excretion are not warranted because of the inherent instability of the drug substance.

## *In Vitro* PK Drug Interactions

Regulatory guidance [6,7] recommends that the potential for PK DDIs of novel therapeutics be investigated at the level of metabolic enzymes and drug transporters. Typically, the potential for DDI is first investigated *in vitro* and/or *in silico*. If positive, the result is followed up in a clinical DDI study to ascertain clinical relevance.

A drug in development can be either the victim of DDI—its PK being altered in the presence of another drug—or the perpetrator. To be a victim of DDI, the ON needs to be a substrate for a phase 1 or 2 metabolic enzyme (CYPs, UGTs, SULT) or a transporter. In general, ONs are metabolized by nucleases rather than CYPs and phase 2 conjugation enzymes and are not typically substrates for the main transporter proteins. In addition, cases of clinically relevant protein displacement are rare; this is specifically true for ONs, which do not impact the protein binding of common small molecule drugs [38]. To be a perpetrator of DDI, the ON needs to be a CYP inducer, a CYP inhibitor, or a transporter inhibitor.

ONs, notably those with an anionic backbone, bind nonspecifically to a range of macromolecules. Although nonspecific binding is generally weak, ONs could theoretically occupy the active sites of enzymes and transporters, and interfere with the PK of concomitant medications as perpetrators. Conjugates or components of ON formulations such as LNPs could potentially interfere with metabolism or transport in a similar fashion. As nonspecific binding is driven by the ON backbone, the conjugated moiety, or a formulation component, the potential for DDI is not likely dependent on the ON sequence. Therefore, the Subcommittee advocates a platform-based approach to PK DDI investigations for ONs. Taking this approach, the lack of potential for DDI *in vitro* would be demonstrated at least once for each combination of backbone chemistry, conjugate, and/or formulation. If the outcome is positive, *in vitro* DDI should be investigated for subsequent members of that ON class.

Assuming negative results in these assessments, a paper argument, referencing the results as well as any published literature, could be generated to indicate that such assessments are not warranted for future compounds within the same chemistry platform. This approach has been accepted by multiple regulatory bodies with some of the member companies on the Subcommittee. Because the metabolites of ONs are predominantly short-chain products and any DDI liability should have been covered by assessment of the full-length ON, the assessment of DDI potential of metabolites of ONs is generally not warranted.

DDI can also occur through mechanisms related to either PD or disease, as described by Humphreys *et al.* [45]. For instance, although givosiran does not inhibit CYPs directly, the potential for indirect inhibition through its intended inhibition of heme synthesis was a concern [75]. Similarly, a GalNAc-conjugated ON in clinical trials for the treatment of hepatitis C virus (HCV) was found to be associated with hyperbilirubinemia; the test article was subsequently demonstrated to be an inhibitor of MRP2, which was particularly relevant because HCV is associated with low MRP2 [76]. Based on these examples, the Subcommittee recommends that Sponsors conduct a thorough literature search regarding the potential impact of PD on CYPs and transporters as well as modulation of CYPs and/or transporter expression in the intended clinical indication and perform additional studies as warranted.

The FDA has also expressed concern about the potential of ONs to impact CYP activity by indirect means. Such mechanisms include interference with the synthesis or degradation of heme or the modulation of cytokines. The latter concern stems, at least in part, from the correlation between proinflammatory cytokines and CYP expression as noted for therapeutic proteins [7]. In such cases, standard *in vitro* testing of CYPs may not yield clinically relevant results and alternate approaches, such as treatment of hepatocytes with plasma from test article-treated human blood, may be warranted.

Test concentrations for ONs should not be adjusted based on percent protein binding (unlike small molecules). The high PPB (eg, >90% bound for PS-modified ONs) does not affect the quantitative measurement of test article through commonly used analytical assay formats such as HELISA and LC-MS.

### Metabolism-mediated DDIs

ONs (excluding delivery moieties) are metabolized by endo- and exonucleases, which are expressed ubiquitously in high abundance in tissues and blood. Therefore, in general, ONs have not been found to be substrates of common drug-metabolizing enzymes like CYPs or UDP-Glucuronosyltransferases [77]. As a result, *in vitro* DDI assessments of ONs as substrates of drug-metabolizing enzymes are not warranted [8]. However, substrate determinations may be warranted for conjugated ONs and for novel components of LNP formulations.

To conduct *in vitro* DDI studies of ONs as inhibitors of drug-metabolizing enzymes, appropriate selection of the test system is critical in achieving clinically meaningful results. Kazmi *et al.* recently investigated the *in vitro* inhibition of CYPs and UGTs by phosphodiester- or PS-linked ONs [78,79]. Direct inhibition of almost all CYPs and UGT enzymes was observed with PS ONs when tested in human liver microsomes. However, no direct inhibition was observed when cryopreserved human hepatocytes were used as the test system. This discrepancy is believed to be related to the uptake mechanism of ONs into hepatocytes, which involves endocytosis; only a very small fraction of the compound can escape endosomes into the cytoplasm to interact with drug-metabolizing enzymes that are located primarily in the endoplasmic reticulum. Based on these results, cryopreserved human hepatocytes appear to be a more relevant test system than microsomes for *in vitro* assessments of enzyme inhibition by ONs [80,81]; however, it is important to demonstrate effective cell uptake in the test system.

This Subcommittee is not aware of any positive results using the hepatocyte system and, thus, the DDI risk mediated by CYP inhibition is deemed to be low.

On the other hand, because several ONs have been positive for CYP induction, the Subcommittee recommends continued testing of all new platforms. At least two anti-miR ONs have been associated with post-transcriptional regulation of specific CYPs leading to alteration of their expression [82]. The siRNA portion of patisiran was associated with mild but reproducible induction of CYP2B6 [66]; due to the low induction and limited number of CYP2B6 substrates, no clinical DDI studies were warranted. In addition, both eteplirsen and golodirsen are weak inducers of CYP1A2 [83,84]. For studies to assess CYP induction potential, as with inhibition studies using hepatocytes, effective uptake of ONs into cells used as the test system should be demonstrated, as has been done for genotoxicity test systems [85,86]. It is also important to evaluate the ON concentrations at the end of the incubation to demonstrate the metabolic stability of ONs in the test system. A platform approach might be acceptable as demonstration of both uptake and stability.

### Transporter-mediated DDIs

To date, ONs, regardless of class, chemistry, length, conjugation, etc., have rarely been demonstrated to be substrates of the major families of transporter proteins involved in cross-membrane transport of small molecules [80,87]. Therefore, their absorption, distribution, and excretion are not likely altered in the presence of inhibitors of major transporters. However, at a minimum, the Subcommittee recommends assessment of an ON as a substrate of specific transporters that could potentially be involved in the TD and/or clearance pathway of the ON. For instance, for ONs with substantial renal excretion as unchanged drug, it may be important to determine whether the ON is a substrate of renal transporters [8]. Similarly, *in vitro* DDI studies of a GalNAc-conjugated ON might focus on hepatic efflux transporters, as opposed to hepatic influx, renal, or gastrointestinal transporters. Once the possibility of an ON being a substrate for specific transporters responsible for distribution/clearance has been ruled out, a platform-based approach may be appropriate for subsequent ONs of the platform.

ONs, their conjugates, or formulation components could potentially compete for active sites of transporters by nonspecific binding, thereby causing steric hindrance of the transporters for concomitantly administered small molecule drugs. Some, although limited, reports suggest that certain ONs are indeed inhibitors of drug transporters, although the IC_50_s were far greater than the unbound plasma concentrations of clinical relevance [48,79,88]. Weak inhibition of transporters has been reported for several of the approved ONs: inhibition MATE1 by casimersen [89], inhibition of OCT1 and OATP1B1 by eteplirsen [90], and inhibition of MATE2K by golodirsen [91]. Therefore, assessment of inhibition in the standard panel of transporters is recommended. In addition, Sponsors should consider whether additional transporters should be evaluated based on the known distribution and/or clearance of the ON. For instance, for locally administered ONs, investigation of transporters relevant to the site of administration might be considered.

The test system recommended for influx transport studies is primary human hepatocytes or cells overexpressing specific human transporters. For efflux transport studies, the use of monolayers or sandwich cultures is recommended. Inside-out membrane vesicles are not recommended, as they do not provide the relevant cellular context because ONs are sequestered in the endosomal compartment, cytoplasmic concentrations are low, and a high fraction of the ON is likely bound to intracellular proteins other than the efflux transporter of interest. Therefore, inhibitory concentrations determined using membrane vesicles may be overestimates of the clinically relevant DDI potential.

### Potential for other DDIs

Generally speaking, because of their size and charge state, all ONs need some special mechanism for cellular uptake (eg, receptor-mediated endocytosis). Therefore, it is important to understand whether any concomitantly administered drugs, endogenous compounds, or components in the diet may potentially alter the PK of ONs through competing with or inhibiting the uptake mechanism. For instance, competition for the asialoglycoprotein receptor could lead to its saturation and impact the TD and, thus, systemic adverse effects of GalNAc-conjugated ONs. Therefore, based on the uptake mechanism, the potential for such DDIs should be addressed on a case-by-case basis using tools, including literature searches and/or *in vitro* or *in vivo* evaluations.

It is generally established that DDI due to PPB displacement is of little clinical significance for the majority of drugs at steady state [92]. Although this conclusion is based primarily on the free-drug hypothesis with small molecule drugs, the principles should also apply to ONs. In addition, the binding sites for highly hydrophilic drugs like ONs are likely different from those for hydrophobic small molecule drugs, which further decreases the likelihood of protein binding-based DDIs between ONs and small molecule drugs. To the best of the Subcommittee's knowledge, there have been no reports of protein binding-mediated DDI for ONs either as perpetrators or as victims.

### Timing of *in vitro* DDI studies

The guidances from FDA [7] and EMA [6] recommend performing *in vitro* DDI studies before the product is administered to patients in large-scale (ie, Phase 2) clinical trials and is generally required before starting Phase 3 trials for small molecule drugs. Specific studies may be warranted earlier depending upon the metabolism and excretion pathways of concomitant medications allowed during clinical trials. Although no specific recommendation is available for ONs, the Subcommittee suggests similar timing for the DDI assessment of ONs. The only exception would be if a waiver can be justified for a well-characterized chemistry based on platform data.

## Conclusions

In general, the recommended approach to the nonclinical assessment of PK/ADME for ONs is similar to that for small molecule drugs. However, modifications in the approach are warranted because, compared with small molecule drugs, the ON class of drugs has substantially less chemical diversity, considerably greater consistency and predictability of PK/ADME properties, and far fewer safety concern related to these properties. Adjustments are also warranted to address potential immunogenicity and its impact of PK; in addition, the PK/ADME of any non-ON component of the drug substance needs to be considered.

The timing of studies may differ slightly. For instance, some TD data are generally collected earlier in development; this is often to confirm delivery to the intended pharmacologic target tissue rather than to guide toxicity assessments. The timing of PPB studies is more relaxed; the extent of binding is generally consistent within a chemical class and has limited impact on safety, including DDIs. *In vivo* metabolism assessments may be phased; metabolism is less of a safety issue for ONs than small molecules because reactive metabolites are not generated.

In addition, slight differences in the scope are warranted because of greater consistency of results across ONs as compared with the phenomenal diversity among small molecule classes. For instance, although single-dose IV PK studies may provide value for non-IV routes of administration, they are not necessarily required to determine the absolute bioavailability of non-IV dosed ONs. TD assessments can be limited to certain set of tissues, for example, the target tissues of PD and toxicity as well as liver, kidneys, and other relevant tissues based on the known distribution pattern of ONs in the class and across species for each ROA. Metabolism occurs through nucleases and is typically similar across species; therefore, the likelihood of disproportionate human metabolites is low. In addition, ONs are known to be excreted as either parent or shortmers. These factors reduce the need for radiolabeled ADME and mass balance studies in animals for full characterization of metabolites and clearance pathways and similarly reduce the need for a clinical radiolabeled ADME study and any required nonclinical support thereof.

In fact, due to the consistency of results within each chemical class of ONs, the Subcommittee advocates the use of a platform-based approach similar to that described in the FDA guidance for COVID-19 vaccines [54]. That is, once sufficient data are available for the platform, presentation of these data should be sufficient to support development of additional ONs of the same platform. This approach could apply to *in vitro* studies of protein binding and PK drug interactions (as recommended by Humphreys *et al.* [45] for GalNAc-siRNAs) and stability of full-length parent, and *in vivo* studies such as TD and mass balance studies. In addition, *in vivo* TD of an LNP-formulated ON should suffice to support use of the same LNP for delivery of other ON payloads. In all cases, the platform (ON or LNP) would need to be defined clearly by the Sponsor; in addition, sufficient data must be available to support the assertion of similar physiochemical properties and ADME profiles among ONs of the same platform.

For many aspects of the nonclinical PK/ADME assessment of ONs, certain situations warrant special consideration. The potential for ADA and their impact on PK needs to be assessed; the Subcommittee supports the approach recommended by Henry *et al.* [25]. For conjugated or LNP-formulated ONs, novel (non-ON) components need to be treated as new chemical entities and assessed in a manner similar to small molecule drugs. In addition, adjustments to the testing approach may be needed for LNP-formulated ONs.
